# Perinatal mental health around the world: a new thematic series

**DOI:** 10.1192/bji.2019.32

**Published:** 2020-02

**Authors:** John Cox

**Affiliations:** Emeritus Professor of Psychiatry, Keele University, UK. Email: john6.cox@gmail.com

**Keywords:** Perinatal psychiatry, perinatal, international, postnatal depression

## Abstract

International multiprofessional teams in primary and secondary care have much to teach psychiatrists, researchers and service planners about the perinatal mental health field. This editorial introduces a new series of papers in *BJPsych International* on perinatal mental health around the world.

Politicians, in spite of – or because of – Brexit turmoil have recently held prolonged debates in the House of Commons on perinatal mental illness and early years family support,^1,2^ when they spoke from personal experience and demanded improved services. Perinatal mental health advocacy by international organisations such as the Marcé Society for Perinatal Mental Health and the Global Alliance for Maternal Mental Health, is not only establishing an implementation research base but is also enabling a more universal as well as culture-specific understanding of service and research priorities, as papers in this new thematic series ‘Perinatal mental health around the world’ will illustrate.

## A spotlight on three countries

The papers from India,^3^ Sweden^4^ and Italy^5^ published in this issue provide readers with insights as to why at national and local level the plight and mortality of new parents with mental disorders are so frequently unnoticed. They point out that the human costs to society of this treatment gap are considerable. They suggest caution, however, before assuming that one size will fit all, and illustrate the different professional skill mixes in primary and secondary care across national boundaries – as well as the impact of family support on the need for residential parent and baby units. Curiously, the effect of perinatal mental illness on fathering is only beginning to be fully acknowledged – and research on grandparenting is in its infancy.

Each paper has emphasised the need to develop comprehensive national guidelines and pathways to care across the primary/secondary care boundary – and regard this as a current priority. The absence of access to specialist perinatal psychiatric services is striking.

### India

In India, Ganjekar and colleagues^3^ have drawn attention to the absence of mental health in research priorities for maternal, newborn and child health and nutrition by national funding bodies. They nevertheless underline the truism that pioneer developments such as the mother and baby unit in Bangalore initiated by Prabha Chandra, and the new service developments outlined by her colleagues in Kerala, demonstrate models of care that can move mountains. Their detailed perinatal research priorities are pertinent not just for Indian funding agencies but also the wider international community.

### Sweden

Birgitta Wickberg in Gothenburg, and her colleagues from Uppsala and Stockholm,^4^ describe in their paper from Sweden how they have already pioneered universal primary care screening and a therapy service which incorporates the Edinburgh Postnatal Depression Scale (EPDS) and offers of ‘listening visits’ by trained midwives, nurses and psychologists. They also ask: where are the psychiatrists and where is the tertiary care? Strikingly they remind us that Sweden is now one of the most multiracial countries in Europe and highlight the need for new skills in this transcultural field, which is so politically sensitive.

### Italy

Pietro Grussu in Padua, and his colleagues in Rome and Venice,^5^ remind us of the frequency of the non-psychotic mental disorders of childbirth and draw attention to the present drivers of change in Italy, which include public concern about the number of mothers who kill themselves during pregnancy or in the puerperium, as well as greater awareness of violence by partners or family members. The lack of specialist perinatal facilities is implicit in this paper.

## A call for thinking outside of the box

Taken together, it is hoped that these multiprofessional papers from Europe and India will provoke the reader to think outside the box of narrow specialism and to maintain an integrative focus for clinical work. At the heart of perinatal mental health around the world there are family relationships, fathers as well as mothers – and an infant growing as a person with a name.

The Editor of this Journal will welcome submissions from other countries which will inform multiprofessional teams in primary and secondary care – and which may also implicitly or explicitly redefine and clarify the role of psychiatrists in this burgeoning field.
